# Improving uptake and use of malaria rapid diagnostic tests in the context of artemisinin drug resistance containment in eastern Myanmar: an evaluation of incentive schemes among informal private healthcare providers

**DOI:** 10.1186/s12936-015-0621-7

**Published:** 2015-03-06

**Authors:** Tin Aung, Christopher White, Dominic Montagu, Willi McFarland, Thaung Hlaing, Hnin Su Su Khin, Aung Kyaw San, Christina Briegleb, Ingrid Chen, May Sudhinaraset

**Affiliations:** Population Services International, Yangon, Myanmar; Bill and Melinda Gates Foundation, Seattle, USA; Global Health Sciences, University of California, San Francisco, CA USA; Department of Health, National Malaria Control Programme, Yangon, Myanmar

**Keywords:** Malaria, rapid diagnostic tests, Private providers, Myanmar, Intervention, Incentives, Artemisinin, Drug resistance, *Plasmodium falciparum*, Elimination

## Abstract

**Background:**

As efforts to contain artemisinin resistance and eliminate *Plasmodium falciparum* intensify, the accurate diagnosis and prompt effective treatment of malaria are increasingly needed in Myanmar and the Greater Mekong Sub-region (GMS). Rapid diagnostic tests (RDTs) have been shown to be safe, feasible, and effective at promoting appropriate treatment for suspected malaria, which are of particular importance to drug resistance containment. The informal private sector is often the first point of care for fever cases in malaria endemic areas across Myanmar and the GMS, but there is little published information about informal private provider practices, quality of service provision, or potential to contribute to malaria control and elimination efforts. This study tested different incentives to increase RDT use and improve the quality of care among informal private healthcare providers in Myanmar.

**Methods:**

The study randomized six townships in the Mon and Shan states of rural Myanmar into three intervention arms: 1) RDT price subsidies, 2) price subsidies with product-related financial incentives, and 3) price subsidies with intensified information, education and counselling (IEC). The study assessed the uptake of RDT use in the communities by cross-sectional surveys of 3,150 households at baseline and six months post-intervention (6,400 households total, 832 fever cases). The study also used mystery clients among 171 providers to assess quality of service provision across intervention arms.

**Results:**

The pilot intervention trained over 600 informal private healthcare providers. The study found a price subsidy with intensified IEC, resulted in the highest uptake of RDTs in the community, as compared to subsidies alone or merchandise-related financial incentives. Moreover, intensified IEC led to improvements in the quality of care, with mystery client surveys showing almost double the number of correct treatment following diagnostic test results as compared to a simple subsidy.

**Conclusions:**

Results show that training and quality supervision of informal private healthcare providers can result in improved demand for, and appropriate use of RDTs in drug resistance containment areas in eastern Myanmar. Future studies should assess the sustainability of such interventions and the scale and level of intensity required over time as public sector service provision expands.

## Background

The emergence of drug-resistant *Plasmodium falciparum* malaria threatens to undermine recent and significant gains in global malaria control [1]. Initially identified in western Cambodia, artemisinin-resistant *P. falciparum* malaria has now been documented in Vietnam, Thailand, Laos, and Myanmar [2]. In response, the World Health Organization is now urging member states within the Greater Mekong Sub-region (GMS) to eliminate the parasite entirely.

The first step towards regional elimination is aggressive control of the disease. Within the GMS, Myanmar has the highest prevalence of malaria, and is therefore furthest from accomplishing this pre-elimination stage. Myanmar accounts for the majority of malaria-related morbidity and mortality in the region [3], and despite a recent decline in transmission (Population Services International (PSI) Myanmar, unpublished observations), the country faces serious challenges in achieving elimination [4], including the spread of malaria through outdoor-biting vectors [5], often in hard-to-reach forested areas [6]. In this operational context, it is critical to target interventions to the human reservoir of infection, best done where people with malaria seek care. In Myanmar, the informal private sector healthcare providers play a critical role in healthcare delivery. While informal providers are potentially a serious threat to progress if ignored, they also present important opportunities if properly capacitated.

Informal private providers comprise a heterogeneous group of providers who lack formal training, with differences in regulatory frameworks and services provided [7]. They are often the first point of care for communities in remote rural areas due to their relatively high numbers, low cost, flexible payment arrangements, responsiveness to patients, long opening hours, and close ties to their communities as opposed to formal and public sector providers [8-15]. As their businesses are comparable to privately owned shops, working with these providers to improve the quality of healthcare requires examination of incentives, both in terms of provider supply and patient demand.

In recent years, practices in Myanmar’s informal private sector have been implicated in the development and spread of artemisinin resistance. Specifically, due to the high price of artemisinin-class compounds, studies have found that oral artemisinin monotherapy (oAMT) were commonly sold in the informal private sector in Myanmar, often at partial doses likely due to the high price of artemisinin-class drugs. An analysis of private sector anti-malarial drug importation records in 2011 demonstrated that approximately 1.6 million adult equivalent treatment doses (AETD) of oAMT were being imported into Myanmar each year, primarily artesunate tablets (PSI Myanmar, unpublished observations). Qualitative data from a series of rapid supply chain surveys indicated that treatment blister packs were often cut into two to three partial courses and provided with little or no blood testing to confirm infection: the worst possible scenario for drug resistance development (PSI Myanmar, unpublished observations).

Acknowledging this threat, the Government of Myanmar, under the Myanmar Artemisinin Resistance Containment (MARC) framework for the containment of artemisinin resistance, set the rapid replacement of oAMT in the private sector with quality assured artemisinin-based combination therapy (ACT) as one of its primary objectives [16]. Population Services International (PSI), a large US-based NGO, was charged with facilitating this process, including managing the ACT subsidy, commodity procurement and import, onward sales to national distributors, monitoring of supply chains, supportive communications and medical detailing, and lastly, measuring the impact of the subsidy intervention and associated regulatory enforcement over time utilizing methods from the ACTwatch research initiative [17].

Another major objective of MARC is to strengthen and improve access to and use of early diagnosis and quality treatment [16]. With the support of the Myanmar Ministry of Health, PSI Myanmar began piloting deployment of RDTs in the informal private sector in early 2013. Despite the successful uptake of ACT across the market, concerns over unnecessary and incomplete treatment remained and the Government of Myanmar requested PSI Myanmar to examine the introduction of rapid diagnostic tests (RDTs) to address both issues. Several studies have reported marked declines in the presumptive use of ACT following large-scale deployment of RDTs [18-24].

Although studies have demonstrated correct and safe use of RDTs by community health workers and volunteers [25-28], particularly in sub-Saharan Africa, few studies have examined the acceptance of RDT testing by informal private healthcare providers [29,30], and only one study looked at large-scale introduction in the context of drug resistance containment within the GMS [31]. This programme, which took place in Cambodia, recommended that future RDT subsidies should include a training component that counsels and educates providers about the role and proper use of RDTs. In addition, the programme also suggested that financial incentives might serve as an important strategy to promote RDT use [31]. This study builds upon these findings by offering different incentive structures to informal providers in Myanmar.

The distribution of RDTs with ACT is expected to be feasible on a large scale, particularly since pharmaceutical products in Myanmar are distributed through a highly centralized supply chain. Although the introduction of RDTs to informal private providers in Myanmar can potentially improve treatment practices, little is known on how to motivate these providers to conduct diagnostic testing for malaria. The study team initiated a six township pilot project to evaluate incentive schemes aimed at improving demand and appropriate use of RDTs among priority outlet types.

## Methods

### Description of interventions

The study team developed a pilot intervention targeted at informal providers, investigating three intervention arms with different incentive packages. Arm 1 tested a price subsidy for RDT resupply at approximately $0.18/RDT with resupply given in exchange for used RDTs and a monthly check-in visit. Arm 2 included the price subsidy provided in Arm 1 as well as a financial, product-related incentive in the form of a free RDT kit for every five RDTs purchased. Arm 3 included the price subsidy provided in Arm 1 as well as bi-monthly intensive support visits by PSI health officers, including one-on-one discussions, information, education and communication (IEC) to providers. All three arms were potentially scalable, using existing private sector ACT supply chains, but differed with regard to cost in incentive or personnel.

Prior to initial deployment of RDTs, PSI product promoters (PPs) identified and mapped all outlets supplying anti-malarials in the six townships. PSI enlisted five resupply points in each township. These were relatively big pharmacies in the area, and located in areas where providers could restock their supply of RDTs. Providers in all three arms were trained on RDT use, interpretation and safe disposal. Specifically, in the case of a negative test, providers were trained to refer the patient to the nearest health facility if they were unable to diagnose for other illnesses. In addition, during training sessions, PSI provided a list of supply points in the township, and instructed providers that they may go to any resupply point on the list. The 6-month pilot intervention study included the deployment of RDTs and provider incentives across six townships in Myanmar. RDT demand and use was assessed over the course of six months (April until September 2013).

### Setting

The pilot intervention was implemented in rural areas of Mon and Shan states in eastern Myanmar. Townships were chosen based on areas with similar malaria risk levels, socio-economic status, level of migration, access to roads, population size, male-to-female ratio, and presence of health centres. Each intervention was randomized to two townships in each of the two states, for a total of six townships.

The interventions specifically targeted three types of informal private provider in each township: providers in general retail stores (GRS), itinerant drug vendors (IDVs), and medical drug representatives (MDRs), including informal private pharmacists. These providers were chosen because they represent a significant portion of the market share for ACT. Training on and supply of RDTs may be particularly important for these types of outlets because they are frequently the first point of seeking care in rural Myanmar. GRS are small shops in the communities, typically selling a variety of goods, including non-health-related products. IDVs often serve as the village doctor, with some IDVs travelling to people’s homes for care ranging from maternal and child health services to malaria diagnosis and treatment. While IDVs may not have received formal healthcare training, most have some experience in service delivery. Most IDVs operate from their homes. MDRs are similar to, and sometimes include pharmacists, knowledgeable about basic drugs and treatments, but with little to no formal training.

### Data collection and sampling

This study assessed the impact of the intervention on RDT uptake and quality of malaria care using two data sources: 1) population-based household surveys of the study communities; and, 2) mystery client interviews with enlisted providers. First, the study conducted pre- and post-roll-out cross-sectional household surveys in the six townships to gauge the change in RDT use in the target population. Multi-staged clustered sampling was employed. In urban areas, the clusters were sampled at the level of wards, and in rural areas, clusters were sampled by village track. Simple random sampling without replacement was employed. In total, the study sampled 90 village tracks at baseline and follow up. The study collected baseline data in May 2013 and conducted a follow-up household survey in September 2013 to assess differences in RDT uptake by intervention arm. For survey implementation, all households in the selected townships were enumerated and screened for inclusion. Inclusion criteria were: 1) having a member of the household who had had a fever in the last three weeks and had either taken an anti-malarial drug or had symptoms consistent with malaria [32]; and, 2) living in an area where PSI supplied ACT in private sector outlets and so could also introduce RDTs. In total, 411 households were included at baseline and 421 households were included at end line. The same villages were followed up from baseline to end line. In total, there were 832 fever cases in the baseline and end line surveys.

Once a household was determined eligible for the study, the head of the household answered questions on basic demographic characteristics (including household socio-economic status), recent episodes of fever among household members, anti-malarial drug use, RDT knowledge and use, and if used, where RDTs were obtained and what RDT test results were given. The main outcome of interest from the household survey was the proportion of RDT use (defined as the proportion of RDT use per population treated for malaria or evaluated for fever in the previous two weeks).

Mystery clients visiting a sample of providers were used to assess the quality of RDT use across the study arms. When enlisted in the project, providers agreed to several potential forms of evaluation, including mystery clients. The study used stratified random sampling by intervention arm and provider type (GRS, IDV and MDR). From the list of all 631 providers, the study randomly selected 20 of each type and intervention arm, with the exception of the MDRs where fewer than 20 per cell were available to be enrolled and, therefore, all were included. The final mystery client sample included 171 providers. Each provider was visited once by a mystery client.

**Figure 1:**
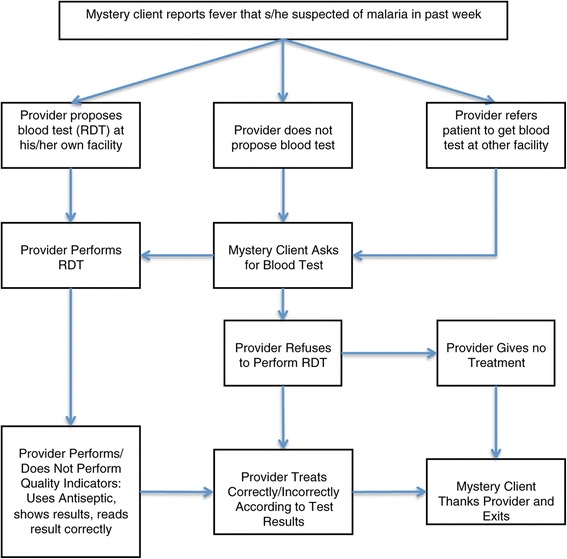
**Mystery client flow chart.**

Mystery clients received intensive training by study personnel on the clinical scenario and completing the assessment form. All mystery clients were malaria negative. The mystery client assessments were conducted between August and September 2013. The mystery client presented at the outlets saying that she/he had fever that she/he thought was like a malaria fever that she/he had experienced on a previous occasion (see Figure 1). The provider could then propose a RDT on site, not propose a RDT at all, or propose a RDT at a different location. If the provider did not spontaneously propose a RDT at his/her own shop, the mystery client was trained to ask for a RDT (i.e. gave a prompt to continue the evaluation). The researcher accompanied the mystery client to the outlet as a friend from town, but did not speak or engage with the provider. The researcher observed the outlet provider and completed the record form after leaving.

The outcome of interest for the mystery client assessment comprised five key steps in conducting and interpreting an RDT for malaria: 1) propose to give RDT test (providers who proposed and performed RDT at their facility); 2) use antiseptic in preparation for finger prick (the RDT kit is supplied with an antiseptic pad inside and could easily be seen as the provider unpacked the kit for use); 3) read the result correctly (providers who accurately read a negative test, assuming that all mystery clients did not have malaria); 4) show the result to the client; and, 5) provide correct treatment according to the result (i.e. providers prescribed ACT for those who read a positive test result; refrained from giving any anti-malarial drugs for a negative client). Results were stratified by intervention arm as well as provider type.

### Analytical methods

All analyses were carried out in STATA 12MP. Analyses weighted the household surveys by population using the *svy* command to account for sample clustering at the village tract level. The study conducted a population average model analysis accounting for clustering at the village level. Multivariable logistic regression was used in order to account for baseline demographic characteristics at the village level and follow up RDT use data at the village level. The outcome of interest was RDT use at follow up measured as a village level indicator (villages could have values from zero to one). Analyses accounting for baseline data were run; however, because there were only 12 individuals who used RDTs at baseline, this variable was omitted from final analyses as it perfectly predicted the outcome of interest. Mystery client data were stratified by provider type and intervention arm. Pearson’s Chi-square was used to detect statistically significant differences for categorical variables. Intent-to-treat analysis was used. That is, providers were analysed according to their intervention arm.

### Ethical considerations

This study obtained ethical approval from the Research Ethics Board (REB) at Population Services International Myanmar and University of California, San Francisco, USA.

## Results

### Description of outlets

Table 1 describes the characteristics of the outlets recruited and trained to provide RDT services. PSI recruited all providers from their ACT programme who agreed to join into the intervention. In total, 631 outlets were trained in RDT use and interpretation across the six townships. GRS comprised the majority of outlets in all three intervention arms, including 53.7% in Arm 1, 73.5% in Arm 2, and 58.8% in Arm 3. Overall, 28.1 and 8.7% were IDVs and MDRs, respectively.

**Table 1:** **The characteristics of outlets recruited and trained for RDT services by study arms**

**Indicators**	**Arm 1**	**Arm 2**	**Arm 3**	**Total**
Total number of RDT outlets	214	264	153	631
General retail store (%)	53.7	73.5	58.8	63.2
Itinerant drug vender (%)	37.9	20.8	26.8	28.1
Medical drug representative (%)	8.4	5.7	14.4	8.7

### Description of household members at baseline

Table 2 describes the demographic characteristics of each intervention arm at baseline. The household survey found that at baseline the majority of participants had primary school of higher (41.6% had at least primary school, 32.6% finished middle school, 10.7% finished high school, and almost 7% finished college or post-graduate studies). This differed across study arm (p < 0.05). There were no statistically significance differences in the gender breakdown or age of respondent across intervention arms, as approximately half of the respondents were female respondents and the mean age of the respondent was 51.1 years.

**Table 2:** **Demographic characteristics of respondents and household members at baseline**

	**Arm1 (n = 129)**	**Arm 2 (n = 139)**	**Arm 3 (n = 143)**	**Total (N = 411)**	**p-value**
**% Female**	45.7	41.0	46.9	44.5	0.581
**Age (mean)**	53.4	49.6	50.5	51.1	0.123
**Highest education in household**					0.000
**No schooling**	15.5	0.72	10.5	8.8	
**Primary grade**	35.6	43.9	47.6	41.6	
**Middle grade**	36.4	32.4	29.4	32.6	
**High School**	9.3	12.2	10.5	10.7	
**Passed Matriculation**	3.9	5.0	0.7	3.2	
**Post-graduate**	2.3	5.8	1.4	3.2	

### Impact of RDT roll-out by intervention arm: household surveys

Combining baseline and end line surveys, 832 households had fever cases (13.2%). At baseline, there were no statistically significant differences across the three arms in regards to RDT use (Arm 1 = 3.0%, Arm 2 = 2.7%, and Arm 3 = 5.4%; p = 0.061) (see Table 3). However, post-RDT roll-out, those in the Arm 3 catchment area were most likely to receive a diagnostic test when presenting with fever (13.0%) compared to Arm 1 (6.4%). Arm 2 demonstrated similar results to Arm 3 with 11.9% of fever patients receiving a diagnostic test by their providers. The before and after surveys indicate that, combined across all three intervention arms, there was a significant increase in RDT use with fever following deployment of RDTs (10.1%) compared to baseline or pre-RDT roll-out (3.7%) (p <0.001). Next, the study tested bivariate analyses accounting for village-level clusters. Multivariable logistic regression accounting for village-level clusters suggests that Arms 2 and 3 increased the odds of use at follow up compared to Arm 1 (OR = 2.21 and OR = 2.71, respectively); however, the results were not statistically significant (see Table 4). There was no statistically significant power to detect changes by arm across time.

**Table 3:** **Percentage of individuals using RDTs, at baseline and endline**

**RDT Use**	**Arm 1**	**Arm 2**	**Arm 3**	**Total**	**p-value**
Baseline	3.0	2.7	5.4	3.7	0.061
Endline	6.4	11.9	13.0	10.1	0.000

**Table 4:** **Odds of Using RDTs at follow up, controlling for village level factors**

	**Odds ratio (p-value)**	**Adjusted odds ratio (p-value)**
Arm 1 Baseline (reference)		
Arm 2 Baseline	2.06 (0.371)	2.21 (0.332)
Arm 3 Baseline	2.29 (0.281)	2.71 (0.203)
Female (village level)	----	0.30 (0.346)
Age (village level)	----	1.05 (0.422)
Education (village level)	----	1.42 (0.459)

### Quality of RDT use: mystery client findings

In total, mystery clients presenting with fever visited 171 providers, including 63 providers in Arm 1, 55 providers in Arm 2, and 53 providers in Arm 3 (see Table 5). Overall, 57.3% of providers proposed a blood test at his/her facility, 34.5% did not propose a blood test, and 8.2% suggested that clients receive a blood test at another facility. Among providers who did not propose a blood test or proposed the test at another facility, the mystery client was trained to prompt the provider for a blood test. An additional 10.5% of providers performed the test after clients requested it, resulting in 67.8% of all providers performing the RDT, reading the result correctly, and properly treating the client based on the result.

**Table 5:** **The quality of RDT services assess through mystery clients by the study arms**

**Indicators**	**Arm 1**	**Arm 2**	**Arm 3**	**Arm 1 vs. Arm 2 Chi2, p-value**	**Arm 1 vs. Arm 3 Chi2, p-value**	**Arm 2 vs. Arm 3 Chi2, p-value**
	**N (%) (n = 63)**	**N (%) (n = 55)**	**N (%) (n = 53)**			
Proposed to conduct RDT at their own facilities (without prompting)	32 (50.8)	35 (63.6)	31 (58.5)	0.157	0.405	0.586
Proposed to conduct RDT at other facilities	7 (11.1)	7 (12.7)	0 (0.0)	0.789	0.005	0.005
Providers proposed and performed all 5 steps of RDT unprompted	25 (39.7)	26 (47.3)	18 (34.0)	0.519	0.658	0.226
Providers who agreed to perform RDT after prompting	3 (4.8)	1 (1.8%)	14 (26.4)	0.710	0.003	0.001
Total providers who performed RDT at their facilities (with and without prompting)	35 (55.6)	36 (65.5)	45 (84.9)	0.269	0.000	0.016
Providers performed RDT, read the result correctly and treat properly (with and without prompting)	27 (42.9)	28 (50.9)	39 (73.6)	0.384	0.000	0.012
The percentages below this row were calculated among those who performed RDT with or without prompting						
Providers who used antiseptic while performing RDT	35 (100.0)	34 (94.4)	41 (91.1)	0.071	0.023	0.508
Providers who read result correctly	30 (85.7)	35 (97.2)	44 (97.8)	0.020	0.013	0.842
Providers who showed results to client	30 (85.7)	34 (94.4)	35 (77.8)	0.107	0.273	0.011
Providers who gave correct treatment	28 (80.0)	30 (83.3)	39 (86.7)	0.643	0.329	0.620

Stratifying by intervention arm, Arm 2 was most likely to conduct a RDT at their own facility (63.6 *vs* 50.8% and 58.5% in Arm 1 and Arm 3, respectively). However, providers in Arm 3 were most likely to perform a RDT (either prompted from the mystery client or unprompted) (84.9% in Arm 3 *vs* 55.6 and 65.5% in Arm 1 and Arm 2, respectively). This was statistically significant between Arm 1 *vs* Arm 3 (p = 0.000) as well as between Arm 2 and Arm 3 (p = 0.016). Of the providers who performed a RDT (n = 116), 94.8% used an antiseptic, 94% read the results correctly, 85.3% showed the results to the client, and 83.6% gave a correct treatment.

The main quality outcome of interest was the proportion of providers who performed the RDT, read the result correctly, and provided appropriate treatment (including not prescribing an anti-malarial following a negative test). Arm 3 was significantly more likely to perform all of these activities, with 73.6% of providers compared with 42.9% of providers in Arm 1 (p = 0.000) and 50.9% of providers in Arm 2 (p = 0.012).

## Discussion

The pre-eminence of private informal providers as a point of first treatment for fevers in rural Myanmar makes this study – the first in Myanmar to document introduction of RDTs in the informal private healthcare sector – of immediate relevance to resistance control programmes. Since informal private healthcare providers are often the first point of care for communities in remote rural areas, a better understanding of how to engage and work with these providers has great potential for improving rural health services, and in particular, for assuring uniform adoption of diagnostic and treatment protocols necessary to prevent the development of resistant strains of malaria.

The findings suggest that introduction of RDTs is feasible among informal private healthcare providers with little or no previous experience of RDT use, and that proper advice by these providers to their clients can lead to increased use of RDTs in the nearby community. Of the three study arms, product subsidy combined with IEC (Arm 3) increased RDT use the most, with only slightly lower results from product subsidy combined with an additional financial incentive (Arm 2). Once accounting for village-level clusters, however, the odds of increased RDT use was no longer statistically significant. Triangulated with mystery client data, however, of particular importance in Myanmar, where artemisinin drug resistance is already found, is that providers in Arm 3 were more likely to correctly follow the treatment protocol indicated by the RDT results, refraining from treating fever patients with anti-malarials when tests were negative (73.6% vs 42.9% in arm 1 and 50.9% in arm 2).

The findings on the effectiveness of the IEC strategy are novel, and overcome a challenge to the introduction of RDTs in private retail settings noted by past researchers [33]. Specifically, a review of the introduction of RDTs in Cambodia’s private sector showed that uptake was low, and suggested that financial incentives be incorporated to encourage their use [31]. On the other hand, a review of RDT use in sub-Saharan Africa identified significant challenges in assuring appropriate information dissemination and practice among retail sector providers [34]. As a result, inappropriate and overtreatment is widespread despite roll-out of RDTs to diverse private sector retailors. While similar challenges exist across Myanmar and the GMS, this study demonstrates that when information and training issues are addressed, RDT acceptance and proper use among providers can be achieved. The study also found that when patients asked about RDTs, the IEC strategy was also more likely to increase provider offering of RDT compared to the other arms. This should be explored in more detail in a future study as to exact mechanisms that might explain this finding. Moreover, subsequent uptake among patients can be measured at a population level.

Analysis of aggressive social marketing efforts to support large-scale introduction of oral contraceptives, condoms, water purification tablets, and zinc supplement treatment for childhood diarrhoea have shown market share rates of between 10 and 15% three years after introduction [35]. In this study, researchers used the higher end of the range, 15%, as a benchmark for what success might potentially look like, noting that even life saving treatments only do slightly better than this, and then only rarely.

Based on the results of the study, two immediate actions are recommended: first, the active engagement of the informal private sector in malaria diagnosis, treatment, and control, particularly in forested malaria-endemic regions; and second, the scale-up introduction of RDTs in the informal private sector to both assure proper treatment and reduce the threat of incorrect in inappropriate presumptive treatment with ACT. This scale-up should include an IEC component combined with a financial incentive.

The study nevertheless has a number of limitations. First, the number of individuals reporting RDT use in household surveys was lower than expected. This presented a number of statistical challenges to detect differences across arms. While the study found an increase in odds of RDT use at follow up, once accounting for village-level clusters, the differences were no longer statistically significant. Therefore, the study may not be powered to sufficiently detect differences. A more proper analysis using difference-in-difference analysis should be used to assess for effects of the intervention across arms and pre-post data; however, the study was not sufficiently powered to perform these more complex analyses. Because there were differences in baseline characteristics, such as age of household members and educational level, this may contribute to findings. However, importantly, there were no differences in fever cases at baseline – therefore, the difference in use of RDT across arms at the end of the intervention was not likely to be attributed to baseline fever loads. Moreover, as described in the Results section, intervention arms differed according to the distribution of informal provider types. The study matched townships on a number of community-level characteristics to provide balance across the arms. Enumeration of provider type revealed that Arm 3 had higher percentages of MDRs and GRS compared to Arm 1. A different study using qualitative interviews with the three different provider types suggest that itinerant drug vendors felt more equipped to provide RDTs compared with GRS and MDR [36]. Therefore, the finding that providers in Arm 3 demonstrated the most significant increase in RDT use suggests that the intervention, and not the provider type, influenced changes in provider behaviour. Second, there may be potential variability in the intensity and consistency of the interventions in different areas. A common practice of PSI Myanmar is to provide small tokens of appreciation to their providers; as a result some providers received additional incentives that were not part of the study, such as umbrellas, lamps or jackets. Third, assessments of RDT use in the target area depended on the household surveys, including cross-sectional surveys at two time points. Because these were not longitudinal, the study was unable to determine true changes in individual practices. Fourth, a major limitation in assessing quality of RDT use was that all mystery clients were malaria negative. Thus the study cannot examine the quality of the providers’ service regarding malaria-positive clients. However, mystery clients are considered the ‘gold standard’ of assessing provider quality, and as noted above, the proper adherence to negative test results has been seen as of more concern than positive treatment in past studies.

The study must also be taken in context and be applied to the present situation in Myanmar. Since efforts to control and eliminate malaria in Myanmar are accelerating, and government spending on health is growing, many of those currently identified as informal private providers may in the future become part of formal efforts to scale-up effective community health worker programmes [37]. This may lead to a changing balance between types of health providers as has recently been the case in Cambodia where expanded community health workers diminished the importance of informal private treatment of malaria [38]. While such changes do not seem imminent in Myanmar, attention needs to be focused on where individuals seek healthcare, and interventions should always be targeted accordingly.

Future studies should continue to document the sustained effectiveness of these interventions at both provider-practice and community-use levels, and assess the sustainability of such programmes in the context of expanding public sector service provision.

## Conclusion

Results suggest that subsidized RDTs be provided on scale to informal private providers in Myanmar that are receiving subsidized ACT. Scale-up should include an IEC component combined with a financial incentive, integrating attributes of the two most successful arms of this study.

### Consent

Written informed consent was obtained from the patient for the publication of this report and any accompanying images.
